# Analysis of the Physiological Activities of Scd6 through Its Interaction with Hmt1

**DOI:** 10.1371/journal.pone.0164773

**Published:** 2016-10-24

**Authors:** Pham Thi Kim Lien, Keiichi Izumikawa, Kei Muroi, Kaoru Irie, Yasuyuki Suda, Kenji Irie

**Affiliations:** 1 Department of Molecular Cell Biology, Faculty of Medicine, University of Tsukuba, Tsukuba, Ibaraki, Japan; 2 Global Innovation Research Organization, Tokyo University of Agriculture and Technology, Fuchu, Tokyo, Japan; 3 Live Cell Super-resolution Imaging Research Team, RIKEN Center for Advanced Photonics, Wako, Saitama, Japan; Medical College of Wisconsin, UNITED STATES

## Abstract

Scd6, a yeast homologue of human RAP55, is a component of messenger ribonucleoproteins (mRNPs) that repress translation by binding to translation initiation factors, and also is a decapping activator along with the binding partners Edc3 and Dhh1. Herein, we report that Scd6 is a substrate of the intrinsic protein arginine methyltransferase, Hmt1, in budding yeast *Saccharomyces cerevisiae*. Mass spectrometric analysis revealed that several arginine residues within the Scd6 RGG motif, which is important for mRNA binding, were methylated in Hmt1 dependent manner. Under stress conditions such as glucose starvation, Scd6 localized to cytoplasmic processing bodies (P-bodies) wherein translationally repressed mRNPs and untranslated mRNAs accumulate. Localization of Scd6 to P-bodies was impaired in *hmt1* deletion mutant and in the presence of methylation-deficient substitution of Scd6. In addition, deletion of *scd6* and *dhh1* led to severe synthetic growth defect at high temperature. Methylation-deficient mutation of Scd6 suppressed the phenotypic defects of *scd6 dhh1* double mutant, whereas methylation-mimic mutation did not, suggesting that the arginine methylation might negatively regulate Scd6 function relating to Dhh1. Therefore, the present data suggest that Hmt1-based arginine methylation is required for Scd6 localization and function.

## Introduction

Messenger ribonucleoprotein (mRNP) complexes comprise transcripts and RNA-binding proteins (RBPs) and regulate gene expression. The lifecycle of mRNP includes mRNA transcription, splicing, transport and localization, translation, and degradation. However, the ensuing gene regulatory mechanisms have not been clarified in the analyses of compositions and kinetics of mRNP complexes at each of these steps [1]. In *Saccharomyces cerevisiae* (*S*. *cerevisiae*), regulators of RNA turnover and translation play essential roles in basal mRNA expression levels under conditions of environmental stimulation. Regulatory pathways of cytoplasmic RNA turnover include decapping by Dcp1/Dcp2 decapping complexes followed by 5′–3′ degradation by exonuclease Xrn1, and 3′–5′ degradation by exosome complexes [2–5]. Several studies have focused on mechanisms that regulate translation initiation at the step in which the recruitment of eIF4G/eIF4E cap-binding complexes occurs. These investigations revealed a competition between translation initiation and decapping [6–9]. Moreover, replacement of initiation factors by decapping enzymes requires other RBPs, which are referred to as decapping activators. Several decapping activators such as Dhh1, Scd6, Stm1, and Pat1 were shown to repress translation initiation, thereby enhancing the decapping processes [6, 8, 10]. However, it remains unclear how functions and interactions of these RBPs are regulated.

Actively translating mRNPs associate with polysomes to initiate translation. Various stress conditions, such as glucose starvation and severe heat shock, induce phase transitions of these mRNPs toward the non-translating state and lead to assembly of most mRNPs into cytoplasmic foci such as processing-bodies (P-bodies) and stress granules (SGs) [11]. Proteins required for active translation are found in SGs, whereas P-bodies include protein factors for mRNA decay machinery such as Dcp1/2, Dhh1, Edc3, and Scd6, suggesting functional diversity of these granules as sites of mRNA storage and mRNA degradation [11]. However, mRNA decay occurs without the formation of large P-bodies [12], and P-body core components have been colocalized with various molecules that are involved in biological processes such as DNA replication and PKA signaling [13, 14]. Hence, knowledge of the activities of P-body components may clarify the physiological functions and kinetics of P-bodies.

Scd6 was originally identified as a multicopy suppressor of clathrin deficiency [15] and a decapping component of mRNPs, as indicated by competition with Edc3 to induce decapping enzymes such as Dcp1/2 [16]. Recently, enhanced decapping activity of Scd6 was related to repression of translation initiation through binding to eIF4G [8, 10]. Scd6 and its homologues are highly conserved in eukaryotes, these proteins contain conserved N-terminal Lsm domains, central FDF motifs, and C-terminal RGG (arginine–glycine–glycine) motifs, which are required for the translation repressing functions of yeast Scd6 [17]. The *D*. *melanogaster* homologue Tral has been shown to interact directly with the conserved RNA helicase DDX6, which is known as Dhh1 in yeast [18]. It has been reported that Dhh1 holds decapping and translation repression functions and is localized to P-bodies [6, 10, 18]. However, details of the interactions of Dhh1 and Scd6 and the mechanisms that regulate functions and locations of these P-body components remain unclear.

Previous studies have shown that proteins containing the RGG box are common substrates of protein arginine methyltransferases (PRMTs) [19, 20]. Specifically, arginine residues of RGG boxes can be monomethylated or dimethylated. In particular, type I PRMTs catalyze the formation of monomethylarginines (MMAs) or asymmetric-dimethylarginines (aDMAs), whereas type II PRMTs catalyze the formation of symmetric-dimethylarginines (sDMAs) [21]. Heterogeneous nuclear ribonucleoproteins (hnRNPs) containing N-terminal RNA-binding motifs in conjunction with RGG repeats are major substrates of PRMT1 in yeast and mammalian cells [22]. Recently, arginine methylation has been shown to mediate RNA–protein, DNA–protein, and protein–protein interactions [23, 24], and *S*. *cerevisiae* Hmt1 was identified as the major type I PRMT [25]. Arginine methylation by PRMT1 is critical for the localization of the hRAP55, Scd6 homologue in mammalian cells [26]. Similarly, Hmt1-mediated methylation of arginine residues in several RBPs, such as Npl3 in budding yeast, regulates protein localization and function [27].

In this study, we investigated protein partners of Scd6, and demonstrated associations of Scd6 and Hmt1. Several arginine residues in RGG motifs of Scd6 were methylated in a Hmt1-dependent manner. Moreover, defects in arginine methylation of Scd6 in *hmt1* mutant cells impaired Scd6-targeting to foci that form under conditions of glucose starvation. However, neither P-body formation nor targeting defects in components of P-bodies were significantly perturbed. We also revealed overlapping functions of Scd6 and Dhh1 that are required for P-body formation and cell growth. Moreover, arginine methylation had no effect on cell growth or P-body formation defects in *scd6 dhh1* double mutant cells. However, similar cell growth was not observed at high temperatures, suggesting possible stress-dependent regulation of Scd6 post-translational modification.

## Materials and Methods

### Strains, plasmids, and general methods

*Escherichia coli* DH5α was used for DNA manipulations and the present yeast strains and plasmids are described in S1 and S2 Tables. Cells were grown in yeast extract-peptone dextrose (YPD), synthetic complete medium (SC), and synthetic minimal medium (SD), and in SC media lacking either amino acids or other nutrients (SC–Ura, SC lacking uracil). General procedures were performed as described previously in “Methods in yeast genetics” [28].

### Gene deletion and protein tagging

Gene disruption and insertion were performed using PCR-based gene replacement, as described previously [29, 30].

### Yeast two-hybrid assays

PJ69-4A cells harboring pGBD-SCD6 were transformed using the yeast two-hybrid library. Transformants were then plated on SC-Leu-Trp plates and were incubated at 30°C for 4 days. Plates were replica plated onto SC-Leu-Trp-His plates, SC-Leu-Trp-His plates containing 1-mM 3-aminotriazole (3-AT), and SC-Leu-Trp-Ade plates, and were incubated at 30°C for 3 days. Twenty-three transformants showed the His+ Ade+ phenotype, and corresponding library plasmids were isolated from transformants, and were reassessed for interactions with Scd6. Insert DNAs were sequenced.

To confirm the interactions of Scd6 and Hmt1 that were identified in two-hybrid screening analyses, pGAD-c1-Hmt1 was constructed and used as a prey vector. PJ69-4A cells were then co-transformed with pGBD-c1 or pGBD-c1-Scd6 with either pGAD-c1 or pGAD-c1-Hmt1, and transformants were then spotted onto SC-Leu-Trp (WL), SC-Leu-Trp-His (WLH), and SC-Leu-Trp-His (WLH) plates containing 1 mM 3-AT and were incubated for 3 days at 30°C.

### Western blot analysis

Samples were loaded onto SDS-PAGE or NU-PAGE gels and were then electroblotted onto Immobilon^™^ polyvinylidene difluoride membranes (Merck Millipore, USA). Blots were blocked for 1 h at room temperature with TBS-M buffer containing 20 mM Tris-HCl (pH 7.5), 150 mM NaCl, and 5% non-fat dry milk, and were then incubated with 1:1,000-diluted primary antibodies in TBS-M buffer overnight at 4°C. After three final washes with TBS buffer containing 20 mM Tris-HCl (pH 7.5) and 150 mM NaCl, blots were incubated with secondary antibodies, and were developed using enhanced chemiluminescence detection kits (Merck Millipore, USA).

### Immunoprecipitation of Scd6Flag

Cells were grown in SC-Ura medium at 30°C to mid-log-phase and were harvested by centrifugation. The cells were then resuspended in XT buffer containing 50 mM HEPES-KOH, (pH 7.3), 20 mM potassium acetate, 2 mM EDTA, 0.1% Triton X-100, 5% glycerol protease inhibitors, phenylmethylsulfonyl fluoride (PMSF), aprotinin, and leupeptin. Glass beads were then added and cells were broken by rigorous vortexing at 4°C (4 times at 3,500 rpm for 30 s). Lysates were then centrifuged for 10 min at 15,000 *g* and supernatants were collected. Extracts were incubated with or without 200 μg/mL RNase A (Wako Pure Chemical Industries, Ltd., Japan) at room temperature for 30 min prior to immunoprecipitation.

To immunoprecipitate Scd6Flag, extracts were incubated with anti-Flag antibody coupled to protein G-Sepharose beads (Sigma Aldrich, USA) for 30 min at 4°C. Scd6Flag-bound beads were then washed three times in XT buffer, and the bound material was eluted with elution buffer containing 0.1 mg/mL Flag peptide in XT buffer for 10 min at 4°C. Samples were subjected to SDS-PAGE followed by colloidal Coomasie blue staining (Thermo Fisher Scientific). RNA was isolated from immunopreciptated samples using RNeasy Mini kit (Qiagen) and subjected to UREA-PAGE followed by SYBR-Gold staining (Life Technologies).

### Protein purification and GST pull down assays

GST- and His_6_- fusion proteins were purified from *E*. *coli* strain BL21 (DE3) using glutathione-sepharose beads (GE) and Ni-NTA agarose beads (Novagen), respectively. GST or GST-Scd6-bound beads were then resuspended in HB buffer containing PBS, 0.5% Tween20, 5 mM MgCl_2_, 5 mM 2-mercaptoethanol, 1 mM PMSF, and a protease inhibitor cocktail (Complete EDTA-free, Sigma Aldrich, USA). His_6_-Hmt1 was then eluted from Ni-NTA agarose beads using a high concentration of imidazole. Proteins were dialyzed and concentrated using Amicon Ultra-2-30K (Merck Millipore Ltd.).

His_6_-Hmt1 was incubated with GST or GST-Scd6 immobilized on beads for 3 h at 4°C. After washing 5 times with PBS, beads were boiled in SDS sample buffer and samples were then subjected to SDS-PAGE, followed by staining with Coomassie Brilliant Blue and immunoblotting with anti-His antibody (Sigma Aldrich, USA).

### Microscopy

Cells were cultured until they reached an OD_600_ of approximately 0.6 in appropriate SC medium. Cells were then collected, washed twice in fresh SC medium with or without 2% glucose, and were then resuspended in fresh SC medium with or without glucose followed by incubation at 30°C for 20 min. Cells were harvested, washed again and immediately examined for granule formation using a Keyence BZ-X700 microscope (Keyence Corporation, Japan) at room temperature. Experiments were performed a minimum of three times. Fluorescence images were processed and analyzed for numbers of cells with foci formation and signal intensities. More than 200 cells were counted and percentages of cells with foci formation were calculated. Fluorescence intensities along 5- μm lines were measured using the linescan function in MetaMorph software (Molecular Devices, USA) and averages of more than 10 foci from each observation were fitted to a Gaussian distribution.

### Tandem mass spectrometry (arginine methylation mapping)

Scd6Flag was immunopurified as described above and was then subjected to SDS-PAGE and stained using Colloidal Blue Staining Kits (Thermo Fisher Scientific, CA, USA). Gel bands containing Scd6-Flag were excised and subjected to in-gel digestion with Chymotrypsin (Sequencing Grade, Promega). The resulting peptides were analyzed using a nanoflow LC-MS/MS system with LTQ-Orbitrap hybrid mass spectrometer (model XL, Thermo Fisher Scientific, CA, USA) as described elsewhere [31] with some modifications. The peptide mixture was separated using reverse phase chromatography with a 0%–40% gradient of acetonitrile containing 0.1% formic acid over 80 min at a flow rate of 100 nl/min using a Mightysil-RP-18 (3 μm particle, Kanto Chemical, Osaka, Japan) fritless column (45 mm × 0.150 mm i.d.). Eluted peptides were sprayed directly into a LTQ-Orbitrap hybrid mass spectrometer and raw data were acquired using Xcalibur version 2.0.7 (Thermo Fisher Scientific, USA) and were then converted to MGF files using Proteome Discoverer version 1.3 software (Thermo Fisher Scientific). Database searches were performed using MASCOT version 2.2.07 software and the Uniprot *S*. *cerevisiae* (strain ATCC 204508/S288c) database with the following parameters: fixed modification, carbamidemethyl (Cys); variable modifications, oxidation (Met); maximum missed cleavages, 1; peptide mass tolerance, 20 ppm; MS/MS tolerance, 0.8 Da. Candidate peptides were selected with probability-based Mowse scores that exceed the threshold, indicating significant homology (p < 0.05; score over 20), and were referred to as “hits.” Precursor peptides containing dimethylarginine residues were detected according to mass increases of 28 Da from those of unmodified precursor peptides. MS/MS spectra of dimethylated peptides were manually assigned, and side-chain fragmentation of methylated arginine peptides was detected as a neutral loss in MS/MS spectra to determine symmetric (31.04 Da) or asymmetric (45.05 Da) dimethylarginine levels [26, 32].

### Polysome analysis

Cells were grown until they reached an OD_600_ of approximately 0.8. Then, they were harvested and resuspended in medium with or without glucose for 20 min. Cultures were washed once with lysis buffer containing 80 μg/mL cycloheximide, 200 μg/mL heparin, 10 mM Tris-HCl (pH 7.5), 0.1 M NaCl, 30 mM MgCl_2_, aprotinin and leupeptin and were then resuspended in this lysis buffer. Glass beads were then added, and cells were broken by rigorous vortexing at 4°C (4 times at 2000 rpm for 30 s). Lysates were then centrifuged for 10 min at 15,000 *g* and supernatants were collected. Sucrose gradients of 10%–50% were prepared in solutions containing 1 mM DTT, 50 mM NH_4_Cl, 50 mM Tris-Acetate (pH 7.0), and 12 mM MgCl_2_ using a Gradient Station (Biocomp Laboratories Inc.). Lysates were added to sucrose gradients and were centrifuged for 3 h at 27,000 rpm in a Beckman Coulter centrifuge (Optima L-100K) at 4°C, followed by fractionation using an Incomparable Piston Gradient Fractionator and a Bio-miniUV monitor (Biocomp Laboratories Inc.). OD_254_ values were monitored to represent fractionation results.

### Statistical analysis

Data are presented as means ± standard deviations (SD) of at least three independent experiments. Statistical analyses were performed using student’s t-test or analysis of variance (ANOVA) followed by Tukey’s test and differences were considered significant when p < 0.05.

## Results

### Scd6 interacts with Hmt1

**Fig 1:**
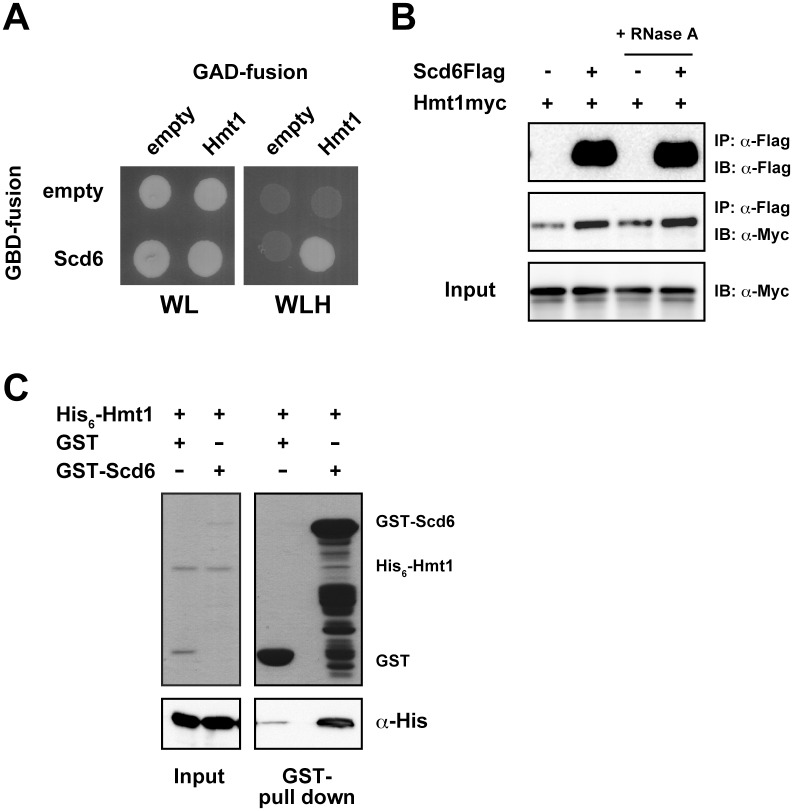
Scd6 interacts with Hmt1. (A) Two-hybrid analysis; The yeast strain PJ69-4A was co-transformed with pGBD-c1 or pGBD-c1-SCD6 with either pGAD-c1 or pGAD-c1-HMT1 and colonies were spotted onto SC-Leu-Trp (WL) and SC-Leu-Trp-His (WLH) plates and incubated for 3 days at 30°C. (B) Co-immunoprecipitation analysis; Scd6Flag was immunoprecipitated from Hmt1myc strains harboring YCplac33-SCD6FLAG or empty vector with an anti-Flag antibody, and were probed for Scd6Flag and Hmt1myc using anti-Flag and anti-myc antibodies, respectively. Similar experiments were performed using total extracts treated with RNase A prior to immunoprecipitation. (C) *In vitro* binding assays; His_6_-Hmt1 was incubated with Glutathione sepharose beads that had been preloaded with equal amounts of GST or GST-Scd6. Recombinant proteins were then detected using Coomassie staining and associated His_6_-Hmt1 was detected using an anti-His antibody.

Scd6 is a translational repressor that reportedly interacts directly with eIF4G to block translation initiation [8]. Scd6 is also known as a decapping activator that contributes to decapping complexes of Scd6, Edc3, Pat1, and Dhh1 [10]. To identify other sets of proteins that associate with Scd6 in cells, we performed yeast two-hybrid screening of yeast cDNA library using Scd6 as bait. In addition to the *DCP1* gene, which encodes a known Scd6-interacting partner, DNA fragments encoding Hmt1 were recovered in these analyses. Other Scd6-interacting candidates are listed in S3 Table. We also detected an interaction between Scd6 and Hmt1 in two-hybrid analyses. Cells expressing both GBD-Scd6 and GAD-Hmt1 exhibited the His+ phenotype (Fig 1A).

Hmt1 is the major type I protein arginine methyltransferase (PRMT) in budding yeast and catalyzes the production of both mono- and asymmetric di-methylarginines on histone and non-histone proteins, especially RNA-binding proteins, and thereby regulates the functions and localizations [20, 27]. Several RNA-binding proteins require mRNAs to associate with other partners in the complex. Thus, to determine whether mRNA is required for the association of Scd6 and Hmt1 *in vivo*, we performed co-immunoprecipitation with or without RNase A treatment (Fig 1B). Immunoprecipitation of Scd6Flag from cell extracts with an anti-Flag antibody led to coprecipitation of Hmt1myc, as detected using an anti-Myc antibody in Western blots. We also found that Hmt1 immunoprecipitated with Scd6 regardless of RNase A treatment, suggesting that these proteins copurify in an RNA-independent manner. Subsequently, we performed *in vitro* binding assays using recombinant proteins to determine whether this interaction is direct or not. In these experiments, His_6_-Hmt1 was significantly bound to GST-Scd6 (Fig 1C), indicating that the Hmt1–Scd6 interaction is direct, and may be akin to enzyme and substrate interactions.

### Scd6 contains asymmetrically methylated arginines in RGG motifs

**Fig 2:**
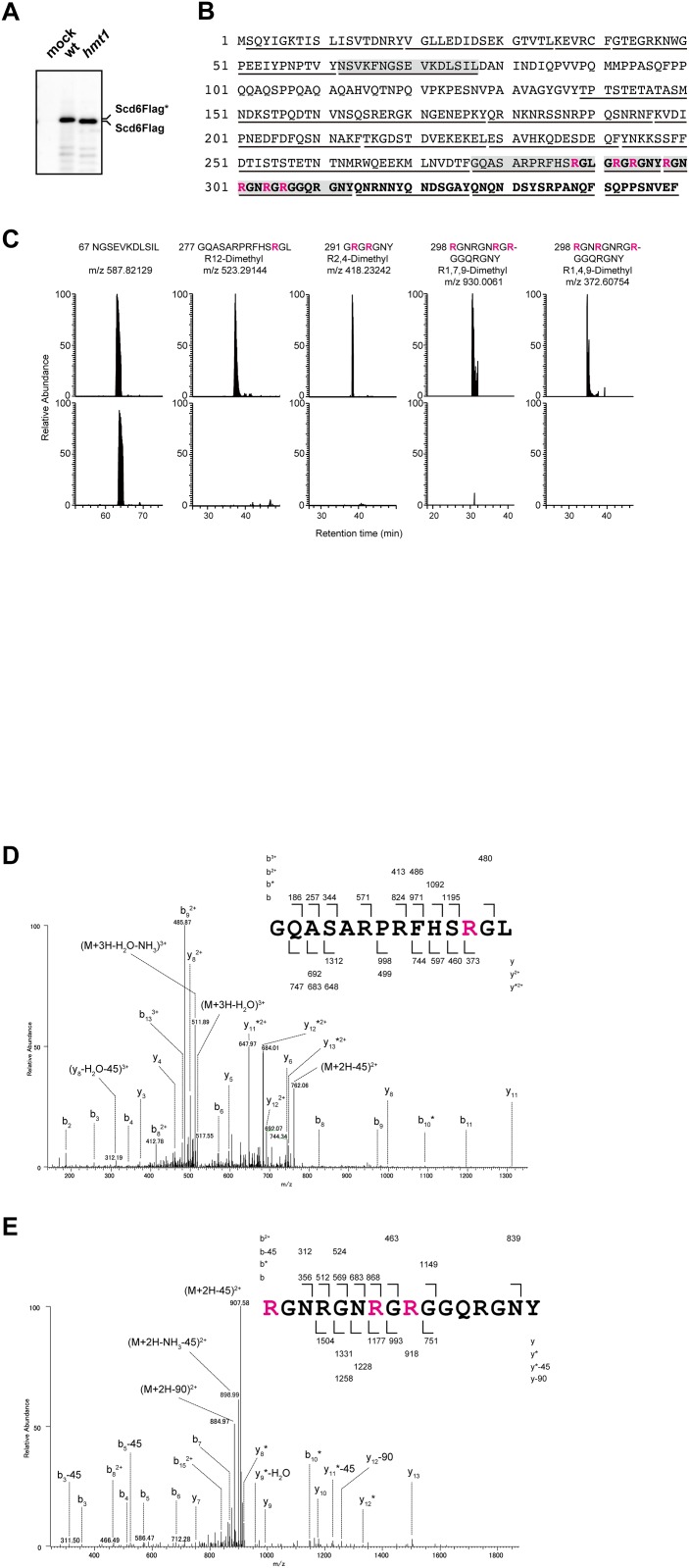
Arginine residues in RGG motifs of Scd6 are dimethylated in a Hmt1-dependent manner. (A) Scd6Flag proteins from wild-type and *hmt1* cells were immunoprecipitated using an anti-Flag antibody and were separated using NU-PAGE gels, and were then visualized by immunoblotting with an anti-Flag antibody. (B) Amino acid sequences of Scd6 are shown and chymotriptic peptides that were identified using Tandem mass spectrometry analyses are indicated by underlines. Red letters indicate asymmetric dimethylarginines and residues of RGG motifs are shown in bold. (C) Chromatograms of four chymotryptic peptides of Scd6 containing asymmetric dimethylarginines were compared between wild-type (upper panel) and *hmt1* cells (lower panel). Signal intensities of peptides were normalized to total ion current chromatograms and ratios of signal intensities of peptides between wild-type and *hmt1* cells are presented as relative abundances of each peptide. NGSEVKDLSIL was used as an unmodified Scd6 peptide. (D, E) MS/MS spectra of identified peptides containing asymmetric dimethylarginines; GQASARPRFHSRGL (D), RGNRGNRGRGGQRGNY (E).

Interactions were observed between Hmt1 and Scd6 in yeast two-hybrid, co-IP, and *in vitro* binding assays, which suggested an enzyme–substrate interaction. PRMTs bind and methylate RGG repeats in numerous RNA-binding proteins, and Scd6 contains RGG repeats in its carboxy-terminus [8, 21]. Accordingly, following immunoprecipitation of Scd6Flag from wild-type and *hmt1* cell lysates using a monoclonal anti-Flag antibody, we found that the Scd6Flag band from *hmt1* cells was slightly smaller than that from wild-type cells (Fig 2A), suggesting that Hmt1 binds and methylates several arginine residues in RGG motifs of Scd6.

To confirm this possibility and determine modifications of Scd6, Scd6Flag from wild-type and *hmt1* cells were immunopurified as described above and were digested with chymotrypsin in gels. Collected peptides were then analyzed using tandem mass spectrometry, and those containing dimethyl arginine were compared. Chymotryptic peptides covering more than 80% of entire protein sequence of Scd6 were detected (Fig 2B). Among these, four peptides contained seven dimethyl arginines in RGG motifs of Scd6 from wild-type cells, but not from *hmt1* cells (Fig 2B and 2C). Peptides containing aDMA or sDMA can be distinguished by neutral losses of dimethylamine (m/z 45.05) and monomethylamine (m/z 31.04), respectively [26, 32]. Thus, we assigned spectra to the peptide 277–290, which contains one aDMA (Fig 2D), and to the peptide 298–313, which contains three aDMAs (Fig 2E). These results suggest that Scd6 is asymmetrically dimethylated in the presence of Hmt1. Note that no significant differences were observed for the associated proteins and RNAs in Scd6Flag-coimmunoprecipitates from wild-type and *hmt1* cells (S1 Fig).

### Hmt1 regulates subcellular localization of Scd6 through arginine methylation

**Fig 3:**
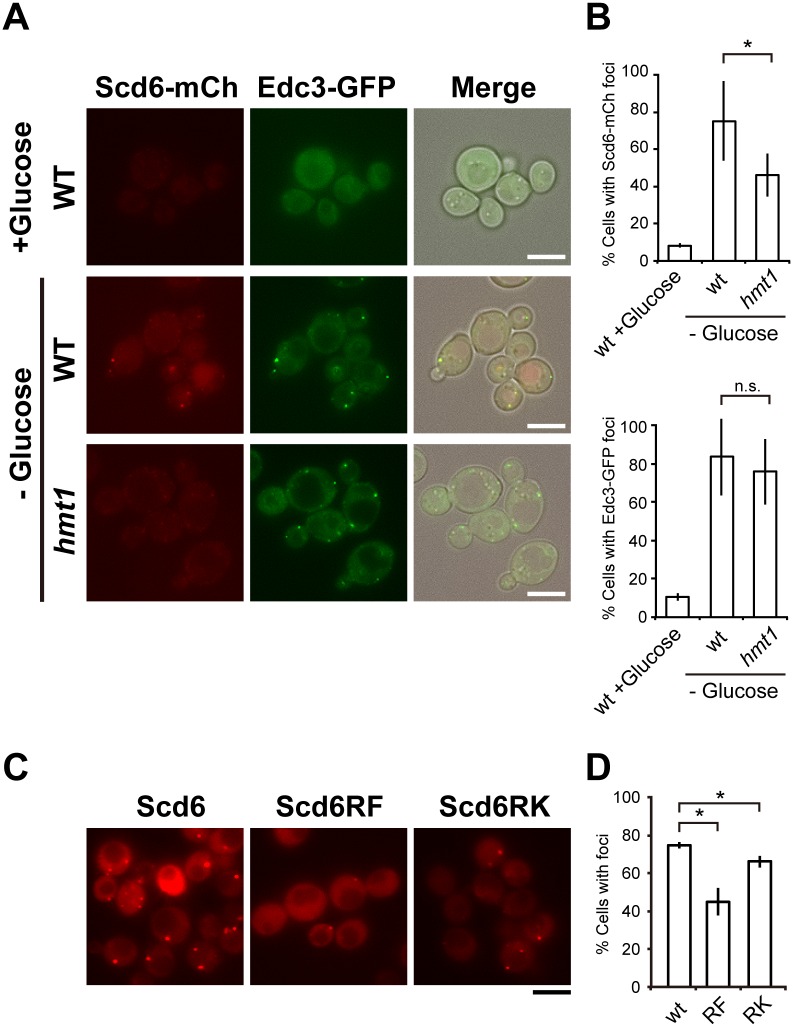
Hmt1 affected Scd6 subcellular localization. (A) Localization of Scd6-mCherry and Edc3-GFP; Wild-type (WT) and *hmt1* mutant cells carrying endogenous mCherry-tagged Scd6 were transformed with the pRS314-EDC3-GFP plasmid. Cells were then grown to mid-log phase and were resuspended in medium lacking glucose; Scale bar, 5 μm. (B) Percentages of more than 200 cells with Scd6-mCherry or Edc3-GFP foci from three independent experiments are presented as means ± standard deviations (SD); *, *P* < 0.05. (C) Scd6-mRFP localization; *scd6* cells containing pRS316-SCD6-mRFP, pRS316-SCD6RF-mRFP, or pRS316-SCD6RK-mRFP were grown and observed as in A. (D) Percentages of more than 200 cells with Scd6-mRFP foci from three independent experiments are shown as means ± SD; *P < 0.05.

Previous studies have shown that Npl3 and Hrp1 are involved in the export of bulk mRNA from the nucleus and that Hmt1 is important for efficient export of these hnRNP complexes [27]. Scd6 is a decapping activator and is accumulated in P-bodies under starvation conditions [8]. Thus, we determined whether Scd6 localization is perturbed in *hmt1* cells under conditions of glucose deprivation. Scd6 was chromosomally tagged with the red fluorescent protein mCherry in its C-terminus and colocalization with the P-body marker Edc3-GFP was observed in wild-type and *hmt1* cells. We confirmed that Scd6-mCherry is still functional using *scd6 dhh1* double mutant (data not shown). In wild-type cells, Scd6-mCherry foci were clearly observed upon glucose deprivation and colocalized well with Edc3-GFP. In contrast, disruption of *HMT1* significantly decreased foci formation of Scd6-mCherry without affecting Edc3-GFP foci formation (Fig 3A and 3B). These data suggest that Hmt1 is required for targeting of Scd6 to P-bodies under stress conditions.

Arginine methylation by PRMTs is implicated in nucleocytoplasmic shuttling of some hnRNPs [20], warranting further detailed analyses of the effects of arginine methylation on Scd6 dynamics in granules. Observed methylated arginine residues in Scd6 were substituted with lysine (methylation deficient, Scd6RK) or phenylalanine (methylation mimic, Scd6RF) [33], and were then visualized by tagging mRFP under glucose-deficient conditions. Under these conditions, the percentage of the cells with Scd6RK-mRFP foci was decreased in comparison with those with Scd6WT-mRFP foci, and this was consistent with the defects observed in *hmt1* mutant cells (Fig 3C and 3D). However, more severe defects of foci formation were observed with Scd6RF-mRFP. These results show that Hmt1-dependent arginine methylation within the RGG motifs of Scd6 is crucial for Scd6 accumulation in P-bodies under glucose-deprivation conditions.

### Scd6 is important for P-body accumulation

**Fig 4:**
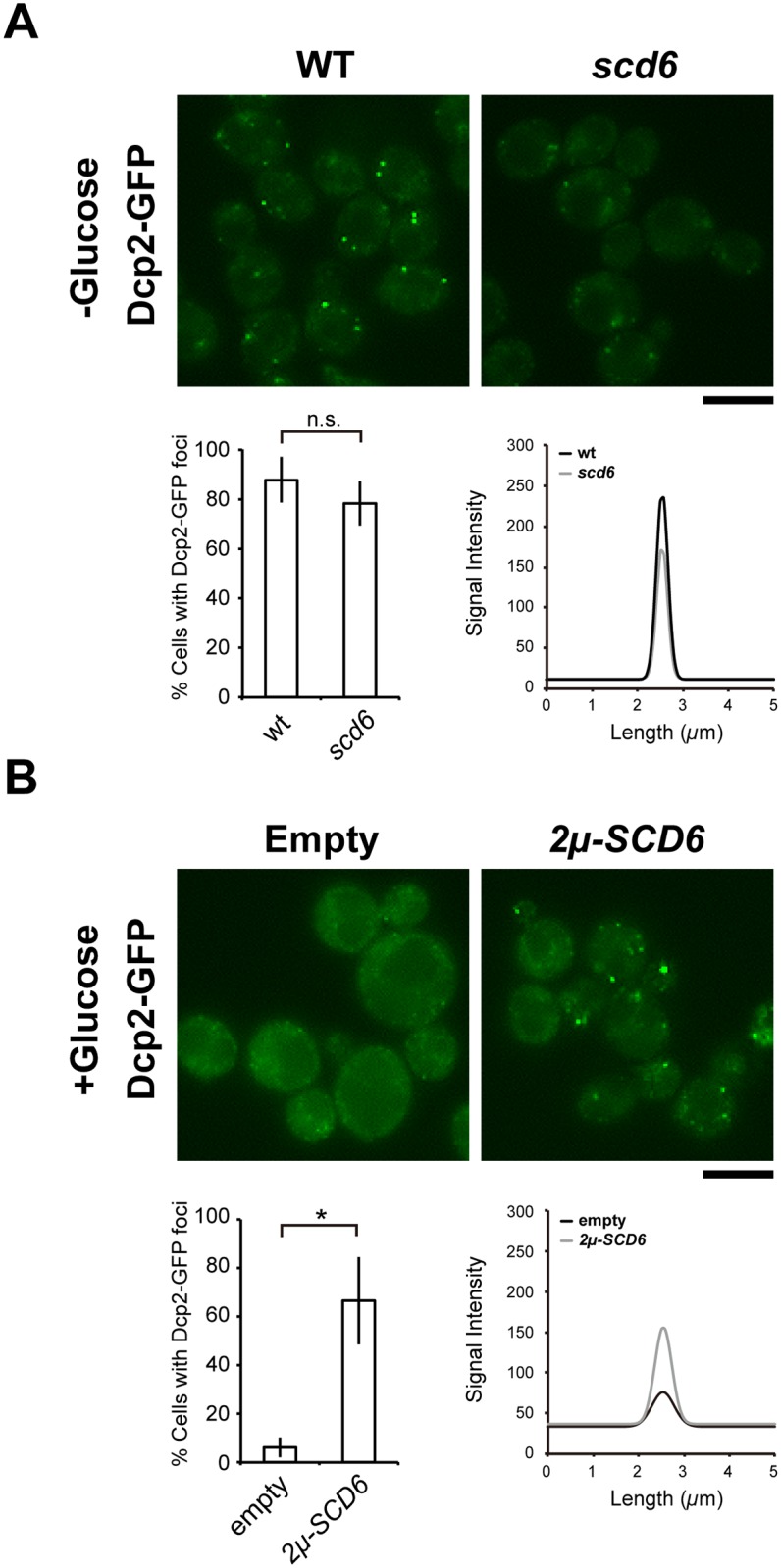
Scd6 plays roles in the accumulation of P-bodies. (A) Foci formation of Dcp2-GFP under glucose depletion condition. Wild-type (WT) and *scd6* mutant cells carrying endogenous Dcp2 tagged with GFP were grown to mid-log phase and were resuspended in medium lacking glucose (-Glucose); Scale bar, 5 μm. Percentages of cells with Dcp2-GFP foci among more than 200 cells from three independent experiments are shown as means ± SD. Fluorescence intensities of Dcp2-GFP were measured using the linescan function in MetaMorph, and fluorescence profiles are shown in the right panel. (B) Foci formation of Dcp2-GFP in Scd6–overexpression condition. Dcp2-GFP cells harboring YEplac195 (2*μ*) or YEplac195-SCD6 (2*μ*-*SCD6*) plasmids were grown to mid-log phase in glucose-containing medium (+Glucose) as shown in A; *P < 0.05.

Scd6 was identified as a core component of P-bodies, along with Dhh1, Dcp2, and Edc3 [10], and associations of these components are reportedly essential for P-body accumulation. Previous studies have shown that cells lacking Dhh1 have defective P-body formation [10]. Although percentages of cells with Dcp2-foci in *scd6* mutant cells under glucose starvation conditions did not differ from those of wild-type cells, signal intensities of these granules in *scd6* cells were lower than those in wild-type cells (Fig 4A). Detailed analyses of wild type cells using linescan showed that foci with strong signal intensities were successfully formed under glucose starvation conditions, but were significantly decreased in *scd6* cells (*P* < 0.01, 0.68-fold). Additionally, Scd6 overexpression induced multiple distinct signals for P-bodies without inducing stress (*P* < 0.01, 9.32-fold) compared to the cells with empty vector (Fig 4B). In contrast with a previous study [10], overexpression of *SCD6* using the *2μ*-plasmid backbone did not lead to growth phenotypes (S2 Fig). These results indicate that Scd6 plays important roles in the accumulation of P-bodies.

### Scd6 and Dhh1 have synthetic effects on cell growth and P-body formation

**Fig 5:**
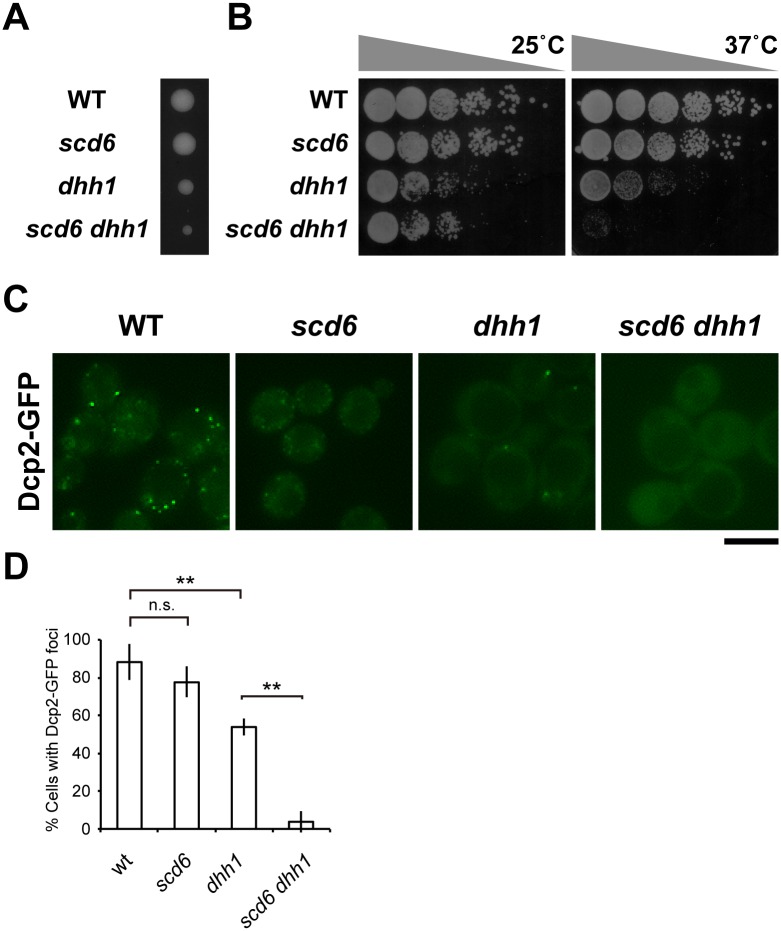
Scd6 and Dhh1 have overlapping functions in cell growth and P-body formation. (A) Growth of *scd6 dhh1* mutant strain; Heterozygous strains carrying mutations in *dhh1* and *scd6* alleles were sporulated, and tetrads were dissected onto YPD medium. Growth after 4 days at 25°C is shown. (B) Growth assays; Wild-type (WT), *scd6*, *dhh1*, and *scd6 dhh1* mutant cells were spotted onto YPD medium and incubated at 25°C or 37°C. (C) Dcp2-GFP foci formation; Wild-type (WT), *scd6*, *dhh1*, and *scd6 dhh1* cells in which endogenous Dcp2 was tagged with GFP were grown in glucose-containing medium to the mid-log phase and were then resuspended in medium lacking glucose; Scale bar, 5 μm. (D) Percentages of more than 200 cells with Dcp2-GFP foci from three independent experiments are shown as means ± SD; **P < 0.01.

Analyses of Scd6 physiological functions *in vivo* were restrained because the *scd6* mutation did not affect growth under normal conditions. Thus, we performed genetic analyses to investigate synthetic functions of Scd6 with components of decapping activators. In these analyses, genetic interactions were identified between *scd6* and *dhh1* alleles. *dhh1 scd6* double mutants showed synthetic growth defects compared with those observed in *dhh1* single mutants at 25°C (Fig 5A), and these growth defects became more severe at 37°C (Fig 5B), suggesting functional redundancies of Scd6 and Dhh1 for cell growth.

As we and others have shown that both Scd6 and Dhh1 have function, in as much as some extent, for P-body formation [7], *scd6 dhh1* double mutant showed almost complete defect for the formation of P-bodies under glucose starvation conditions (Fig 5C and 5D). These data confirm the shared roles of Scd6 and Dhh1 that were observed in *in vivo* experiments, including roles in P-body formation under stress conditions. Previously, global translation repression and P-body formation were correlated under conditions of glucose deprivation [7]. Accordingly, if Scd6 acts as a general translation repressor, defects in translation repression in *scd6 dhh1* double mutants are likely correlated with defects in P-body formation. As a model of global translational repression, glucose deprived cells exhibit declines in translation with polysome profiles [7, 9]. In the present study, wild-type and *scd6 dhh1* cells were cultured in glucose-containing medium until the mid-log phase and were then subjected to glucose deprivation prior to polysome analyses. Under glucose starvation conditions, *scd6 dhh1* cells showed similar reductions in polysome fractions to those observed in wild-type cells (S3 Fig), suggesting that overlapping cell growth and P-body formation functions of Scd6 and Dhh1 may not be related to global translation repression.

### Arginine methylation regulates Scd6 function in relation to Dhh1

**Fig 6:**
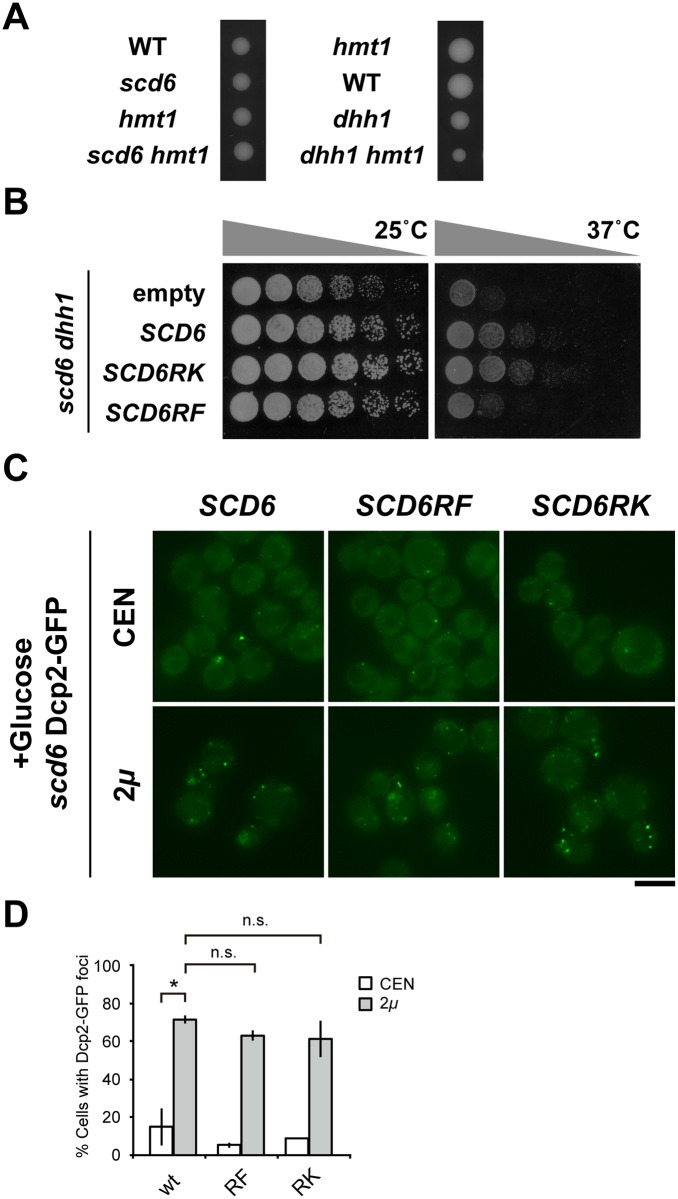
Hmt1 regulates Scd6 function. (A) Growth of *scd6 hmt1* and *dhh1 hmt1* mutant strains. Strains that were heterozygous for *scd6 hmt1* and *dhh1 hmt1* were sporulated, and tetrads were dissected onto YPD medium. Growth was determined after 4 days at 25°C. (B) Growth assays; *scd6 dhh1* mutant cells harboring empty vector (empty) or plasmids containing wild-type, methylation-deficient (*SCD6RK*), or methylation-mimic (*SCD6RF*) substitutions were spotted onto YPD medium and incubated at 25°C or 37°C. (C) *scd6* Dcp2-GFP cells harboring YCplac33 (CEN) or YEplac195 (2*μ*) plasmids containing wild-type (*SCD6)*, methylation-deficient (*SCD6RK*), or methylation-mimic (*SCD6RF*) substitutions were grown to mid-log phase in glucose-containing medium (+Glucose); Scale bar, 5 μm. (D) Percentages of Dcp2-GFP foci among more than 200 cells from three independent experiments are shown as means ± SD; *P < 0.05.

Since the *scd6 dhh1* double mutants showed synthetic growth defects as described above, we next investigated the genetic interaction between *dhh1* and *hmt1*. Tetrad analysis revealved that *dhh1* single mutant grows slower than wild-type, and *dhh1 hmt1* double mutant grows much more slower than *dhh1* single mutant, similarly to *scd6 dhh1* mutant. *hmt1*, *scd6* and *scd6 hmt1* mutants showed normal cell growth (Fig 6A). Thus, Hmt1 and Scd6 may regulate Dhh1-mediated cell growth in similar manners.

We determined whether Hmt1-dependent arginine methylation of Scd6 is involved in Scd6 and Dhh1 mediated regulation of cell growth. In these experiments (Fig 6B), expression of wild-type Scd6 recovered cell growth in *scd6 dhh1* mutant cells at 25°C. Moreover, suppressive effects of Scd6 methylation-mimic and -deficient substitutions were indistinguishable from wild-type cells at physiological temperature. In further experiments at 37°C, both wild-type and methylation-deficient Scd6 suppressed cell growth, whereas methylation-mimic Scd6 did not.

Under normal growth conditions, overexpression of methylation-mimic and -deficient Scd6 induced the formation of P-bodies to similar degrees as overexpressed wild-type Scd6 (Fig 6C and 6D). No detectable differences in foci formation of Edc3-GFP or Dcp2-GFP were observed in *hmt1* mutant cells compared with that in wild-type cells under glucose starvation conditions (Fig 3A and S4 Fig). Thus, our observations suggest that although arginine methylation of Scd6 by Hmt1 is essential for targeting of Scd6 to P-bodies, it is not required for cooperative contributions of Scd6 and Dhh1 to the formation of P-bodies under physiological and stressed conditions, such as glucose starvation. However, further studies are necessary to determine the involvement of methylation in the regulation of Scd6 function following acute stress conditions such as heat shock.

## Discussion

Post-translational modifications are key regulators of signal transduction, protein–protein and RNA–protein interactions, and of protein localizations. Herein, we showed that Scd6 associates directly with Hmt1, the budding yeast homologue of human type I PRMT. Mass spectrometrical analysis revealed that several arginine residues within the RGG motif of Scd6 are methylated in a manner dependent on Hmt1. Arginine-methylation is required for P-bodies targeting of Scd6, which is a decapping activator that functions in part with Dhh1.

P-body localization of Scd6 has been shown under conditions of glucose deprivation [8], and previous studies suggest that P-bodies contain multiple proteins of mRNA decay [34]. The present data show that Hmt1 binds and methylates Scd6. Hmt1 was previously shown to catalyze arginine methylation of mRNP components with RGG motifs such as Npl3, Sbp1, and Ded1, which are reportedly localized in RNA granules [21]. In addition, the P-body component Ebs1 [35] was recovered in our experiments, confirming the specificity of our screening methods, and Ebs1 was also recovered in proteomic analysis of Hmt1-TAP associating proteins [36]. These observations suggest the presence of a functional link between Hmt1 and components of P-bodies [37].

Several arginine residues within the RGG motif of Scd6 were methylated in a Hmt1 dependent manner. Moreover, mass spectrometry analyses revealed the presence of aDMAs in Scd6 peptides from wild-type cells, and these modifications were not observed in samples from *hmt1* mutant cells. Although Scd6 was not recovered in proteomic analyses using a methylarginine antibody, the present data show heavy modifications of this protein in wild-type cells. However, neither PRMT1 nor PRMT5 were recovered as interacting partners for Scd6-homologs in mammalian cells, and only the aDMA modification was detected *in vitro* [26]. Hence, future assessments of degrees of methylation in individual RGG-motif containing proteins and identification of responsible enzyme(s) may reveal functional and dynamic roles of modifications of RGG-motif containing mRNP components.

Depletion of Hmt1 resulted in a specific targeting defect of Scd6 under conditions of glucose depletion, whereas remarkable foci formation defects were not followed for other components of P-bodies such as Dcp2 and Edc3. Similarly, methylation of mammalian RAP55A by PRMT1 specifically regulated localization to P-bodies without perturbing P-body formation or integrity [26]. Thus, the regulatory mechanism for P-body targeting of Scd6 homologs following PRMT1-dependent arginine methylation is widely conserved from yeast to mammals. In the present experiments, Scd6 and Dhh1 additively promoted P-body formation independently of Hmt1-mediated arginine methylation. Hence, mechanisms that lead to Scd6 accumulation in P-bodies, and their regulation by Hmt1-dependent arginine methylation, remain uncharacterized. Recent accumulating evidence indicates that Hmt1 methyltransferase activity is fundamental to protein functions and various molecular processes. In particular, following interactions with Hmt1, Ccr4-Not complexes were involved in mRNA maturation and Npl3-dependent nuclear export, and likely participated in cell-cycle progression by stabilizing cyclin mRNA in response to environmental stimuli [38, 39]. Previous data suggest that Scd6 is functionally involved in the activities of these Hmt1-targets. In particular, Scd6 and Npl3 played similar roles in the targeting of eIF4G for translation repression [8], and *scd6 ccr4* mutant cells had more severe synthetic growth defects than *ccr4* mutant cells (data not shown). Further studies are required to characterize the involvement of Scd6 in nuclear export of mRNA, and to determine whether Hmt1 mediates the effects of Scd6 on cell cycle progression as it does for Npl3 and Ccr4-Not.

Methylation-deficient mutation of Scd6 led to reduced granular-targeting of proteins compared with wild-type proteins. However, methylation-mimic substitutions resulted in similar effects. These substitutions may affect protein structure or associations of Scd6 with other components such as Dhh1. Previously, PRMT1 depletion increased the association of RAP55A and RCK/p54 (the homolog of Dhh1) [26]. Likewise, the present methylation-mimic Scd6 did not suppress synthetic growth defects in *dhh1 scd6* mutant cells at elevated temperature (37°C), whereas both methylation-deficient Scd6 and wild-type Scd6 did. Although, in the present experiments, we were unable to identify specific conditions that affect the methylation status of Scd6, our observations may suggest that stress-dependent regulation of Scd6 is due to Hmt1-mediated methylation. Further analysis of post-translational modifications of mRNP components and the corresponding regulatory roles under various stress conditions are required to elucidate the functions and the structure of granules within cells.

Previous studies suggest that arginine methylation of RNA-binding proteins by PRMTs can modulate binding affinities for RNAs, rather than protein–protein interactions of targets [20, 39]. However, no relevant changes in protein–protein interactions or RNA species were associated with the expression of Scd6Flag in wild-type and *hmt1* cells. Thus, arginine methylation of Scd6 may contribute to mRNA target specificity rather than to general associations of Scd6 with RNAs or protein partners. However, to validate these hypotheses, specific mRNA targets of Scd6 need to be identified, and the effects of Hmt1-mediated arginine methylation on specificity need to be analyzed. A set of Scd6-precipitated mRNAs was submitted to the Gene Expression Omnibus (GEO) by Dan Klass in 2010. These data show that mRNAs that are transcribed from sub-telomeric regions, and coding mRNAs for proteins of telomere capping maintenance and/or DNA replication, were substantially accumulated in Scd6-precipitates. Because proteins for DNA replication stress responses could also be targeted to P-bodies, it will be of interest to determine whether Hmt1 regulates Scd6 targeting to P-bodies under these stress conditions.

In summary, we provide evidence of important roles of Scd6 with Dhh1 in the formation of P-bodies and cell growth, particularly under stress conditions. Specifically, Hmt1 associates with Scd6 to facilitate arginine methylation, leading to efficient targeting of Scd6 to P-bodies. Whether the present post-translational modifications, such as arginine methylation, offer common mechanisms that facilitate the dynamics of mRNP components under stress conditions remains to be clarified.

## Supporting Information

S1 Fig No remarkable changes were observed for Scd6Flag associated proteins and RNAs in wild-type and *hmt1* cells. (A) Colloidal Blue staining of Scd6Flag immunoprecipitates; Scd6Flag proteins from wild-type and *hmt1* cells were immunoprecipitated using an anti-Flag antibody and were separated using SDS-PAGE gels followed by Colloidal blue staining. (B) Purified RNAs from Scd6Flag immunoprecipitates; Scd6Flag proteins were immunoprecipitated using an anti-Flag antibody. RNA species, which were purified from those Scd6Flag immunoprecipitates, were subjected to UREA-PAGE followed by SYBR-Gold staining.
(TIF)

S2 Fig Scd6 overexpression does not affect cell growth. Growth assays; Cells containing indicated plasmids were spotted onto SC medium lacking uracil (SC-Ura) and were incubated at 30°C.
(TIF)

S3 Fig Scd6 was not essential for general translation repression under glucose starvation conditions. Wild-type (WT) and *scd6 dhh1* cells were grown in rich medium (+Dex) and were subjected to glucose deprivation (-Dex) and typical polysome profiles (OD_254_ traces) are presented. Small and large ribosomal subunits (40S and 60S, respectively), monosomes (80S), and polysomes are labeled.
(TIF)

S4 Fig P-body formation is not affected by *HMT1* discruption. Dcp2-GFP foci formation; Wild-type and *hmt1* cells expressing Dcp2-GFP were grown to mid-log phase and resuspended into medium lacking glucose.
(TIF)

S1 Table Strains used in this study. (PDF)

S2 Table Plasmids used in this study. (PDF)

S3 Table Results of Yeast two-hybrid screening. (PDF)
