# Supplementary figures and images for: Analysis of the Physiological Activities of Scd6 through Its Interaction with Hmt1
Source: PLoS One. 2016 Oct 24;11(10):e0164773. doi: 10.1371/journal.pone.0164773 (PMC5077174; doi:10.1371/journal.pone.0164773)

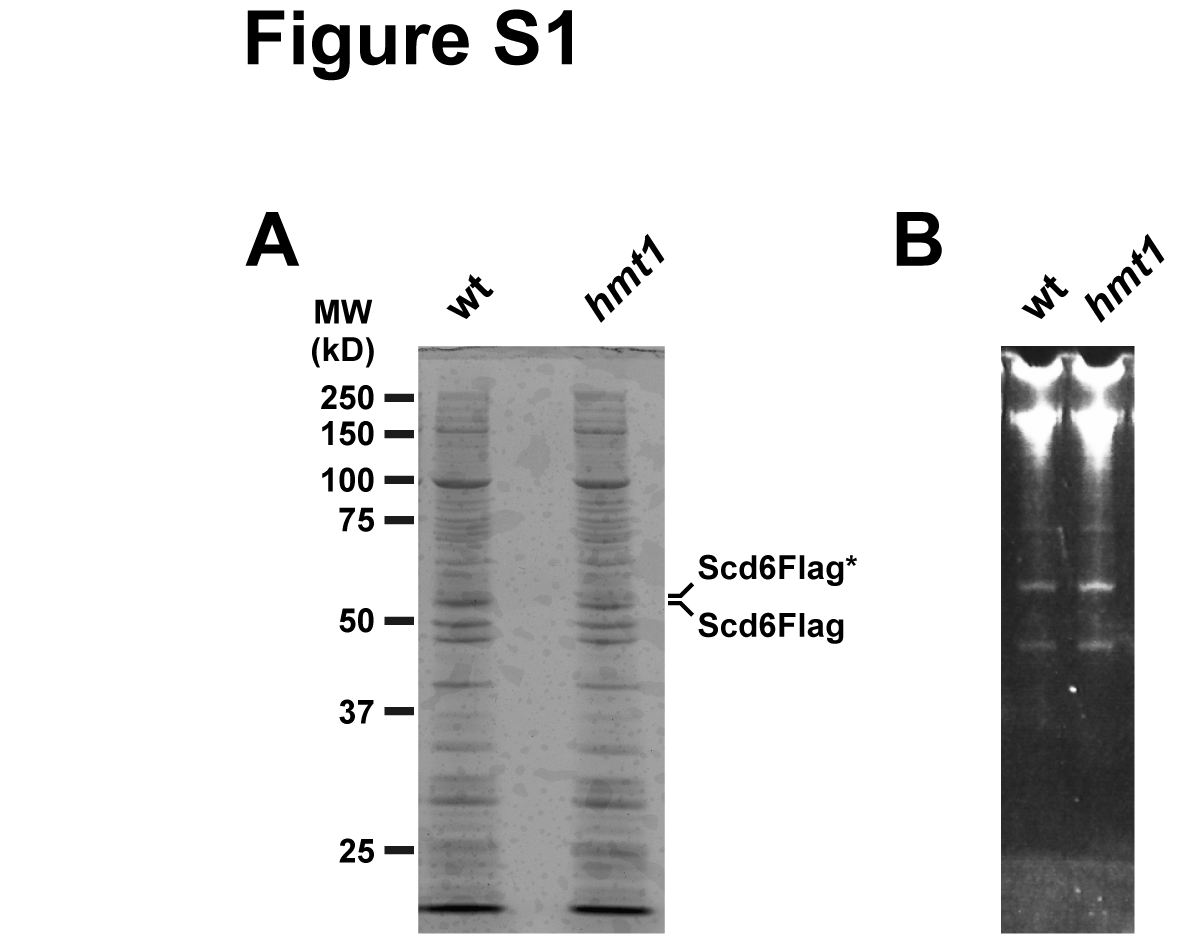

Supplement: S1 Fig — (A) Colloidal Blue staining of Scd6Flag immunoprecipitates; Scd6Flag proteins from wild-type and hmt1 cells were immunoprecipitated using an anti-Flag antibody and were separated using SDS-PAGE gels followed by Colloidal blue staining. (B) Purified RNAs from Scd6Flag immunoprecipitates; Scd6Flag proteins were immunoprecipitated using an anti-Flag antibody. RNA species, which were purified from those Scd6Flag immunoprecipitates, were subjected to UREA-PAGE followed by SYBR-Gold staining. (TIF) [file pone.0164773.s001.tif]

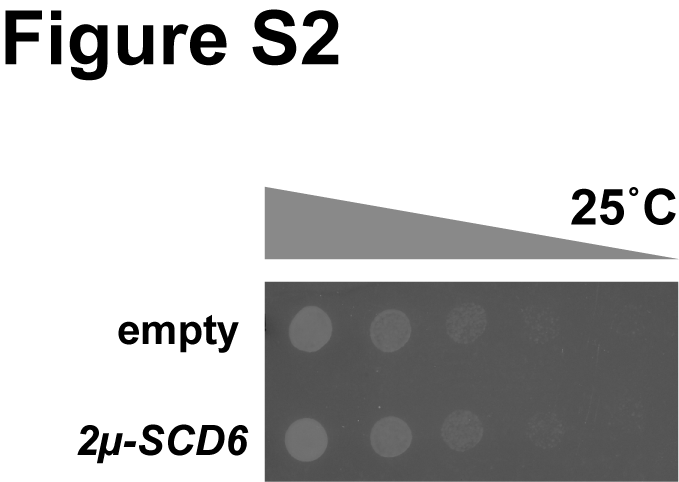

Supplement: S2 Fig — Growth assays; Cells containing indicated plasmids were spotted onto SC medium lacking uracil (SC-Ura) and were incubated at 30°C. (TIF) [file pone.0164773.s002.tif]

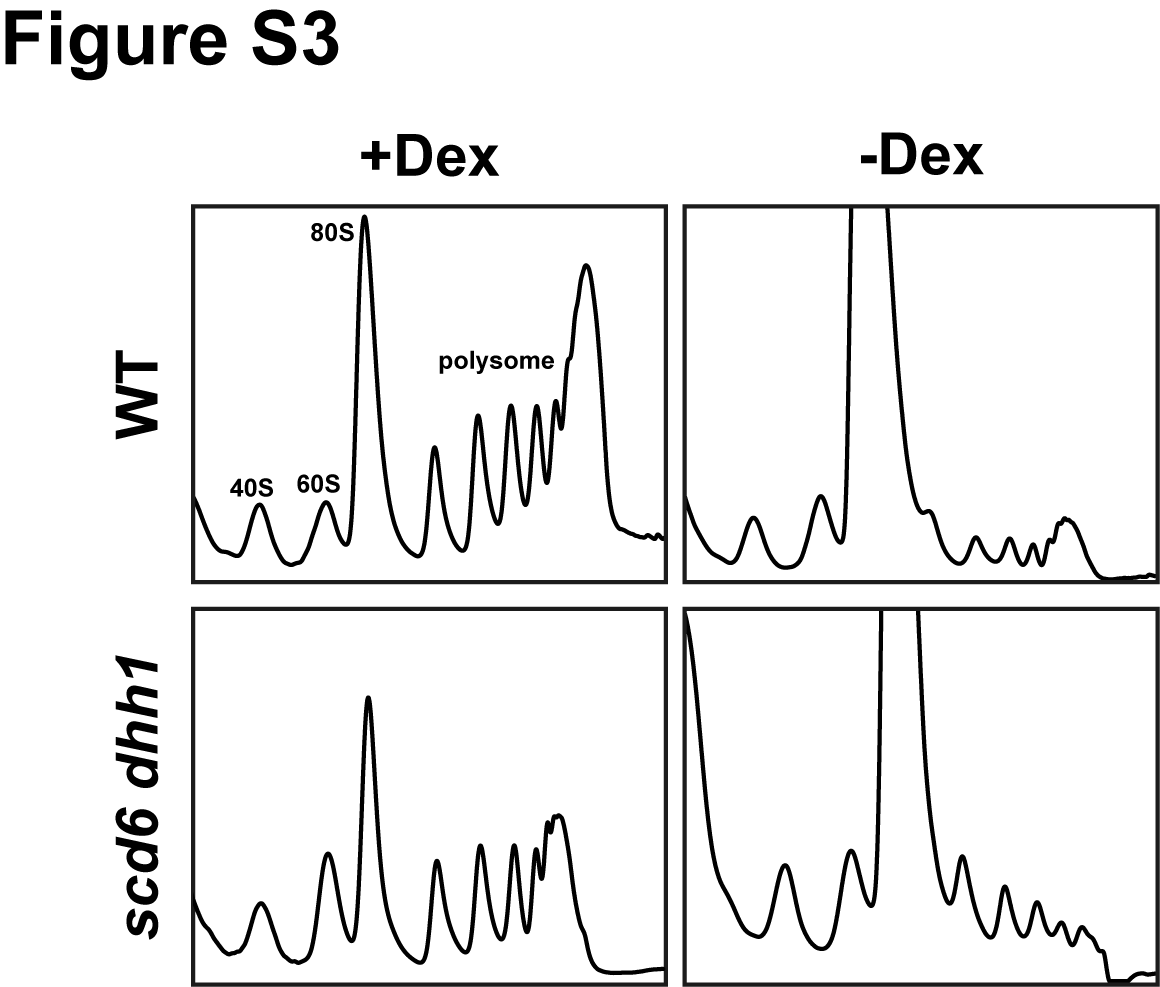

Supplement: S3 Fig — Wild-type (WT) and scd6 dhh1 cells were grown in rich medium (+Dex) and were subjected to glucose deprivation (-Dex) and typical polysome profiles (OD254 traces) are presented. Small and large ribosomal subunits (40S and 60S, respectively), monosomes (80S), and polysomes are labeled. (TIF) [file pone.0164773.s003.tif]

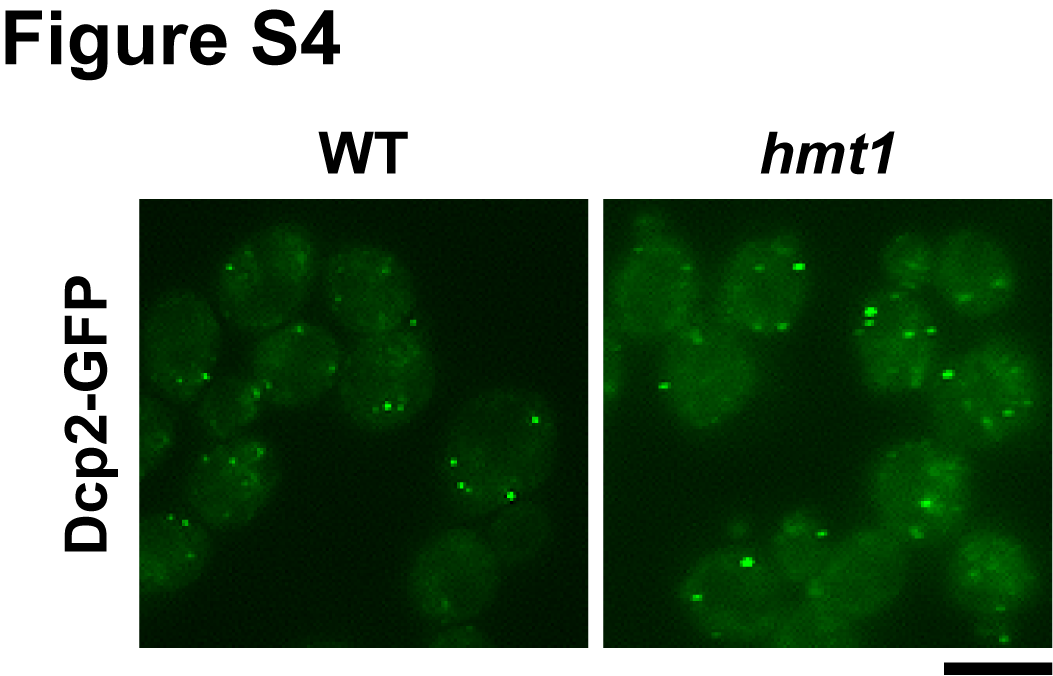

Supplement: S4 Fig — Dcp2-GFP foci formation; Wild-type and hmt1 cells expressing Dcp2-GFP were grown to mid-log phase and resuspended into medium lacking glucose. (TIF) [file pone.0164773.s004.tif]
