# Supplementary material for: Analysis of the Physiological Activities of Scd6 through Its Interaction with Hmt1
Source: PLoS One. 2016 Oct 24;11(10):e0164773. doi: 10.1371/journal.pone.0164773 (PMC5077174; doi:10.1371/journal.pone.0164773)
Supplement: S1 Table — (PDF) [file pone.0164773.s005.pdf]

S1 Table. Yeast strains used in this study.

Name	Genotype	Source
10B	MATα ade2 trp1 can1 leu2 his3 ura3 GAL psi+ HOp-ADE2-HO 3' UTR	[1]
10BD	MAT**a**/MATα ade2/ade2 trp1/trp1 can1/can1 leu2/leu2 his3/his3 ura3/ura3	[1]
10BD-ds	MAT**a**/MATα ade2/ade2 trp1/trp1 can1/can1 leu2/leu2 his3/his3 ura3/ura3 DHH1/dhh1Δ::CgLEU2 SCD6/scd6Δ::CgHIS3	This study
10BD-sh	MAT**a**/MATα ade2/ade2 trp1/trp1 can1/can1 leu2/leu2 his3/his3 ura3/ura3 SCD6/scd6Δ::CgLEU2 HMT1/hmt1Δ::CgHIS3	This study
10BD-dh	MAT**a**/MATα ade2/ade2 trp1/trp1 can1/can1 leu2/leu2 his3/his3 ura3/ura3 DHH1/dhh1Δ::CgLEU2 HMT1/hmt1Δ::CgHIS3	This study
10BD-dsh	MAT**a**/MATα ade2/ade2 trp1/trp1 can1/can1 leu2/leu2 his3/his3 ura3/ura3 DHH1/dhh1Δ::CgLEU2 SCD6/scd6Δ::CgHIS3 HMT1/hmt1Δ::CgTRP1	This study
10BD-s	MAT**a**/MATα ade2/ade2 trp1/trp1 can1/can1 leu2/leu2 his3/his3 ura3/ura3 SCD6/scd6Δ::CgLEU2	This study
s-DCP2	MAT**a**/MATα ade2/ade2 trp1/trp1 can1/can1 leu2/leu2 his3/his3 ura3/ura3 SCD6/scd6Δ::CgLEU2 DCP2/DCP2-GFP::HIS3	This study
d-DCP2	MAT**a**/MATα ade2/ade2 trp1/trp1 can1/can1 leu2/leu2 his3/his3 ura3/ura3 DHH1/dhh1Δ::CgLEU2 DCP2/DCP2-GFP::HIS3	This study
ds-DCP2	MAT**a**/MATα ade2/ade2 trp1/trp1 can1/can1 leu2/leu2 his3/his3 ura3/ura3 DHH1/dhh1Δ::CgLEU2 SCD6/scd6Δ::CgHIS3 DCP2/DCP2-GFP::HIS3	This study
h-DCP2	MAT**a**/MATα ade2/ade2 trp1/trp1 can1/can1 leu2/leu2 his3/his3 ura3/ura3 HMT1/hmt1Δ::CgHIS3 DCP2/DCP2-GFP::HIS3	This study
dh-DCP2	MAT**a**/MATα ade2/ade2 trp1/trp1 can1/can1 leu2/leu2 his3/his3 ura3/ura3 DHH1/dhh1Δ::CgLEU2 HMT1/hmt1Δ::CgHIS3 DCP2/DCP2-GFP::HIS3	This study
10BD-h	MAT**a**/MATα ade2/ade2 trp1/trp1 can1/can1 leu2/leu2 his3/his3 ura3/ura3 HMT1/hmt1Δ::CgHIS3	This study
h-S	MAT**a**/MATα ade2/ade2 trp1/trp1 can1/can1 leu2/leu2 his3/his3 ura3/ura3 HMT1/hmt1Δ::CgHIS3 SCD6/SCD6-mCherry::natNT2	This study
10BD-HMT1	MAT**a**/MATα ade2/ade2 trp1/trp1 can1/can1 leu2/leu2 his3/his3 ura3/ura3 HMT1/HMT1-13myc::kanMX6	This study
s-1	MAT**a** ade2 trp1 can1 leu2 his3 ura3 scd6Δ::CgLEU2	This study
10B-S	MATα ade2 trp1 can1 leu2 his3 ura3 GAL psi+ HOp-ADE2-HO 3' UTR SCD6-mCherry::natNT2	This study
h-1	MAT**a** ade2 trp1 can1 leu2 his3 ura3 hmt1Δ::CgHIS3	This study
ds-1	MAT**a** ade2 trp1 can1 leu2 his3 ura3 dhh1Δ::CgLEU2 scd6Δ::CgHIS3	This study
10B-DCP2	MATα ade2 trp1 can1 leu2 his3 ura3 GAL psi+ HOp-ADE2-HO 3' UTR DCP2-GFP::HIS3	This study

s-DCP2-1	*MAT***a** *ade2 trp1 can1 leu2 his3 ura3 scd6Δ::CgLEU2 DCP2-GFP::HIS3*	This study
d-DCP2-1	*MAT***a** *ade2 trp1 can1 leu2 his3 ura3 dhh1Δ::CgLEU2 DCP2-GFP::HIS3*	This study
ds-DCP2-1	*MAT***a** *ade2 trp1 can1 leu2 his3 ura3 dhh1Δ::CgLEU2 scd6Δ::CgLEU2 DCP2-GFP::HIS3*	This study
h-DCP2-1	*MAT***a** *ade2 trp1 can1 leu2 his3 ura3 hmt1Δ::CgHIS3 DCP2-GFP::HIS3*	This study
dh-DCP2-1	*MAT***a** *ade2 trp1 can1 leu2 his3 ura3 dhh1Δ::CgLEU2 hmt1Δ::CgHIS3 DCP2-GFP::HIS3*	This study
HMT1-1	*MAT*α *ade2 trp1 can1 leu2 his3 ura3 HMT1-13myc::kanMX6*	This study
PJ69-4A	*MAT***a** *trp1-901 leu2-3,112 ura3-52 his3-200 gal4D gal80D LYS2::GAL1-HIS3 GAL2-ADE2 met2::GAL7-lacZ*	[2]

1. Tadauchi T, Matsumoto K, Herskowitz I, Irie K. Post-transcriptional regulation through the HO 3'-UTR by Mpt5, a yeast homolog of Pumilio and FBF. The EMBO journal. 2001;20(3):552-61. doi: 10.1093/emboj/20.3.552. PubMed PMID: 11157761; PubMed Central PMCID: PMC133468.
2. James P, Halladay J, Craig EA. Genomic libraries and a host strain designed for highly efficient two-hybrid selection in yeast. Genetics. 1996;144(4):1425-36. PubMed PMID: 8978031; PubMed Central PMCID: PMC1207695.
