# Supplementary material for: Analysis of the Physiological Activities of Scd6 through Its Interaction with Hmt1
Source: PLoS One. 2016 Oct 24;11(10):e0164773. doi: 10.1371/journal.pone.0164773 (PMC5077174; doi:10.1371/journal.pone.0164773)
Supplement: S2 Table — (PDF) [file pone.0164773.s006.pdf]

**S2 Table. Plasmids used in this study.**

| Name | Description | Source |
|---|---|---|
| YCplac33 | *URA3, CEN-ARS* | [1] |
| YCplac33-SCD6 | *URA3, CEN-ARS, SCD6* | This study |
| YCplac33-SCD6RK | *URA3, CEN-ARS, SCD6RK* | This study |
| YCplac33-SCD6RF | *URA3, CEN-ARS, SCD6RF* | This study |
| YCplac33-SCD6FLAG | *URA3, CEN-ARS, SCD6FLAG* | This study |
| YEplac195 | *URA3, 2μ* | [1] |
| YEplac195-SCD6 | *URA3, 2μ, SCD6* | This study |
| YEplac195-SCD6FLAG | *URA3, 2μ, SCD6FLAG* | This study |
| YEplac195-SCD6RK-FLAG | *URA3, 2μ, SCD6RK-FLAG* | This study |
| YEplac195-SCD6RF-FLAG | *URA3, 2μ, SCD6RF-FLAG* | This study |
| pGBD-c1 | *TRP1, 2μ, ADH1pr-GAL4-BD* | [2] |
| pGBD-c1-SCD6 | *TRP1, 2μ, ADH1pr-GAL4-BD-SCD6* | This study |
| pGAD-c1 | *LEU2, 2μ, ADH1pr-GAL4-AD* | [2] |
| pGAD-c1-HMT1 | *LEU2, 2μ, ADH1pr-GAL4-AD-HMT1* | This study |
| pCgLEU2 | PCR template, *C. glabrata LEU2 in pUC19* | [3] |
| pCgHIS3 | PCR template, *C. glabrata HIS3 in pUC19* | [3] |
| pCgTRP1 | PCR template, *C. glabrata TRP1 in pUC19* | [3] |
| pFA6a-13myc-kanMX6 | PCR template, *13myc-ADH1t::kanMX6* | [4] |
| pRS314-EDC3-GFP | *TRP1, CEN-ARS, EDC3-GFP* | This study |
| pRS316-SCD6-mRFP | *URA3, CEN-ARS, SCD6-mRFP* | This study |
| pRS316-SCD6RF-mRFP | *URA3, CEN-ARS, SCD6RF-mRFP* | This study |
| pRS316-SCD6RK-mRFP | *URA3, CEN-ARS, SCD6RK-mRFP* | This study |
| pGEX-5X-1 | *GST-fusion expression vector* | GE Healthcare |
| pGEX-5X-1-SCD6 | *GST-SCD6* | This study |

| | | |
|---|---|---|
| pET28a (+) | *$His_6$-fusion expression vector* | Novagen |
| pET28a-HMT1 | *$His_6$-HMT1* | This study |


1. Gietz RD, Sugino A. New yeast-Escherichia coli shuttle vectors constructed with in vitro mutagenized yeast genes lacking six-base pair restriction sites. Gene. 1988;74(2):527-34. Epub 1988/12/30. PubMed PMID: 3073106.
2. James P, Halladay J, Craig EA. Genomic libraries and a host strain designed for highly efficient two-hybrid selection in yeast. Genetics. 1996;144(4):1425-36. Epub 1996/12/01. PubMed PMID: 8978031; PubMed Central PMCID: PMCPMC1207695.
3. Sakumoto N, Mukai Y, Uchida K, Kouchi T, Kuwajima J, Nakagawa Y, et al. A series of protein phosphatase gene disruptants in Saccharomyces cerevisiae. Yeast (Chichester, England). 1999;15(15):1669-79. Epub 1999/11/26. doi: 10.1002/(sici)1097-0061(199911)15:15<1669::aid-yea480>3.0.co;2-6. PubMed PMID: 10572263.
4. Longtine MS, McKenzie A, 3rd, Demarini DJ, Shah NG, Wach A, Brachat A, et al. Additional modules for versatile and economical PCR-based gene deletion and modification in Saccharomyces cerevisiae. Yeast (Chichester, England). 1998;14(10):953-61. Epub 1998/08/26. doi: 10.1002/(sici)1097-0061(199807)14:10<953::aid-yea293>3.0.co;2-u. PubMed PMID: 9717241.
