# Supplementary material for: Analysis of the Physiological Activities of Scd6 through Its Interaction with Hmt1
Source: PLoS One. 2016 Oct 24;11(10):e0164773. doi: 10.1371/journal.pone.0164773 (PMC5077174; doi:10.1371/journal.pone.0164773)
Supplement: S3 Table — (PDF) [file pone.0164773.s007.pdf]

S3 Table. Result of Yeast two-hybrid screening

Gene	Description
HSE1	Subunit of the endosomal Vps27p-Hse1 complex
GYP1	*cis*-Golgi GTPase-activating (GAP) protein for yeast Rabs
RPS28A	Protein component of the small (40S) ribosomal subunit
RPS28B	Protein component of the small (40S) ribosomal subunit
ISF1	Serine rich, hydrophilic protein
DCP1	Subunit of Dcp1p-Dcp2p decapping enzyme complex
HMT1	Arginine methyltransferase
EBS1	Protein involved in translation inhibition and nonsense-mediated decay
UTP11	Subunit of U3-containing Small Subunit (SSU) processome complex
HSP82	Hsp90 chaperone
